# Catheter-Related Candida Endocarditis on the Right Atrial Septum – A Case Report

**DOI:** 10.7759/cureus.2158

**Published:** 2018-02-05

**Authors:** Waliul Chowdhury, Muhammad Uzair Lodhi, Intekhab Askari Syed, Umar Rahim, Maxwell Miller, Mustafa Rahim

**Affiliations:** 1 Medical Student, Department of Medicine, Raleigh General Hospital, Beckley, Wv; 2 Pre-Medical Student, Department of Sciences, Queens University of Charlotte, Nc; 3 Medical Student, Department of Medicine, Lincoln Memorial University-Debusk College of Osteopathic Medicine; 4 Assistant Clinical Professor of Internal Medicine, West Virginia University School of Medicine

**Keywords:** infective endocarditis, candida albicans, catheter related infection, atrial septum

## Abstract

The use of subcutaneous catheter devices has increased over the past two decades along with its associated infections. One of the complications is infective endocarditis (IE), which usually occurs on the valves of the heart. However, IE can rarely occur on the atrial septal wall. The most common pathogens associated with catheter-related IE are staphylococcus bacteria, and it is rarely caused by fungi. We present a case of a 75-year-old Caucasian female with infective endocarditis located on the right side of the atrial septum, caused by Candida albicans due to the use of a subcutaneous catheter port. We will discuss the diagnostic criteria and treatment plan for this patient and other treatment options available for these cases. To our knowledge, a similar case was reported in Brazil, but this is the first reported case in the United States of catheter-related infective endocarditis of the right atrial septal wall due to Candida albicans.

## Introduction

Infective endocarditis is described as an infection of the endothelial lining of the heart. Native or prosthetic valves are usually involved, but it can also involve nearby structures or cardiac devices [1]. Right-sided endocarditis is rare due to lower hemodynamic pressures, but the risk increases significantly in patients using central venous catheters [2]. The diagnosis of IE can be easily established in patients with classic features, such as fever, new cardiac murmur, or bacteremia. However, in clinical practice, atypical presentations occur frequently, which makes the diagnosis more difficult. Therefore, the diagnosis of IE should depend on integrating multiple factors, such as clinical, microbiological, imaging, and laboratory findings [1]. The treatment for cases with no valvular involvement is still not completely understood [2]. We present a case of a 75-year-old Caucasian female with infective endocarditis located on the right side of the atrial septum, caused by Candida albicans.

## Case presentation

History and physical examination

The patient is a 75-year-old Caucasian female, who was feeling unwell a day before coming to the hospital. According to her family, she was febrile with a temperature 103 F. She could not provide much information. Most of her information was obtained from past medical records. She was afebrile during initial presentation. She did not mention any chest pain, pressure, or heaviness. She denied any nausea, vomiting, or diarrhea, upper respiratory symptoms, sore throat, or earaches. Her past medical history consisted of the following: recurrent urinary tract infection, multidrug resistant infections, and colonization with extended spectrum beta lactamase (ESBL) infection, previous Clostridium difficile colitis infection, recent acute kidney injury, non-insulin-dependent diabetes mellitus, hypertension, encephalopathy, functional quadriplegia, history of an old cerebrovascular accident (CVA) with deficit, chronic diastolic heart failure, septic arthritis (treated years ago), anemia, coronary artery disease (CAD), rheumatoid arthritis (RA) with deformity, osteomyelitis of the elbow, and hyperlipidemia. Her past surgical history included a cholecystectomy, surgery of both knees, heart bypass surgery, and cardiac catheterization. The patient lived a very sedentary lifestyle and required assistance in the activities of daily living.

On physical examination, the patient was in slight distress. Her vitals were as follows: blood pressure of 103/64, pulse of 77 beats per minute, respiratory rate of 20 breaths per minute, and temperature of 103 F.

The patient had bilateral arcus senilis. The head was atraumatic and normocephalic. Pupils were equal, round, and reactive to light and accommodation. There was no pallor or icterus. The ears were remarkable. The throat was within normal limits. There was a carotid bruit on neck examination. The neck was supple with no lymphadenopathy or thyromegaly. S1 and S2 were normal but there was a systolic ejection murmur, unchanged from a previous exam at the right sternal border. Respiratory sounds were clear to auscultation with no wheezes, crackles, or bronchial breath sounds. The stomach was distended but bowel sounds were audible. There was no visceromegaly. There were chronic changes in both extremities but the absence of Osler nodes or Janeway lesions. Peripheral pulses were somewhat depressed. The patient had deformities of rheumatoid arthritis but no symptoms of acute inflammatory arthritis. The patient had contractures of both lower and upper extremities. There were speech impairment and diffuse muscle weakness. Deep tendon reflexes were depressed. Cranial nerve testing was significant for right-sided facial asymmetry.

Hospital course

Considering the fever and new heart murmur in this patient, blood cultures, urine cultures, complete blood count, metabolic panel, and urinalysis were ordered for the patient. Her blood cultures grew Candida in both bottles. Subsequently, her subcutaneous port device was immediately removed and a two-dimensional (2D) echocardiogram was ordered. Given her family’s request, past medical history, and the location of the vegetation, surgical intervention was not performed.

**Figure 1:**
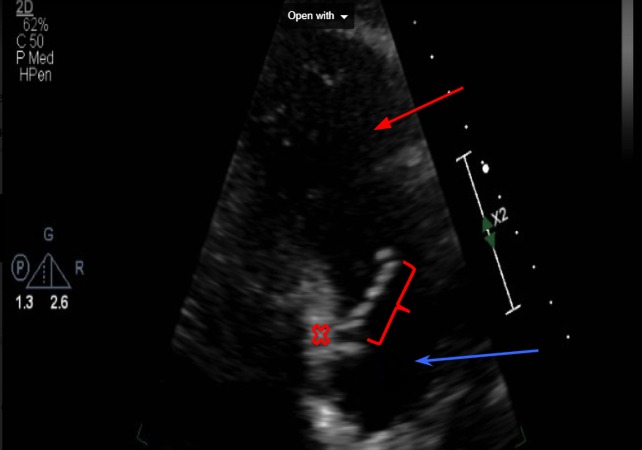
Right-sided 2D echocardiogram showing the vegetation adhered to the right interatrial septum Red bar = vegetation, red x = interatrial septum, blue arrow = right atrium, red arrow = right ventricle 2D: two-dimensional

**Figure 2:**
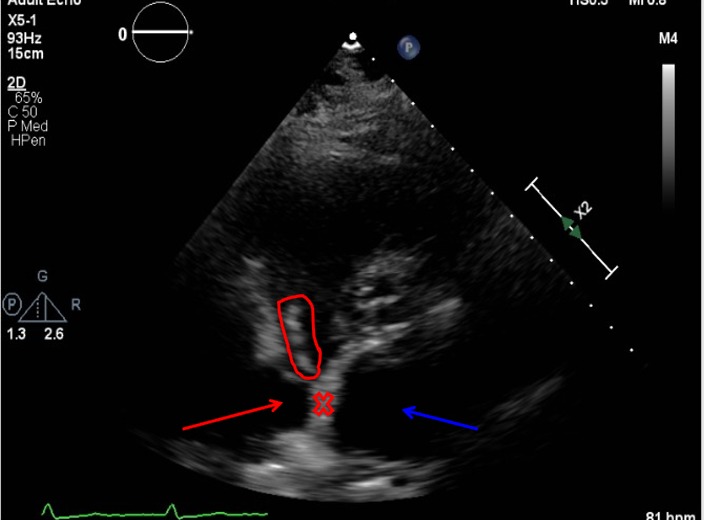
Two-chambered 2D echocardiogram showing the vegetation on the right side of the interatrial septum Red circle = vegetation, red x = interatrial septum, red arrow = right atrium, blue arrow = left atrium 2D: two-dimensional

In addition to blood cultures being positive for Candida albicans, urine cultures were also positive for gram-negative bacilli. The hematology results showed a significantly high white blood cell (WBC) count with neutrophil predominance, as shown in table 1. The coagulation results are shown in Table 2. The metabolic panel is shown in Table 3. The urinalysis results are shown in Table 4. The 2D echocardiogram results showed vegetation on the right side of the interatrial septum, as labeled in Figures 1-2 below. Considering a recent acute kidney injury in our patient, amphotericin b was avoided and the patient was started on caspofungin for the initial treatment, followed by lifelong suppression with fluconazole.

**Table 1:** Hematology results

Collected	Result	Units	Reference Range
White blood cells	14.7	X10^3	4.8-10.8
Hemoglobin	11.8	X10^6	12.0-16.0
Mean corpuscular hemoglobin concentration	31.8	g/dl	33.0-37.0
Red cell distribution width	17.2	%	11.5-14.5
Neutrophils	89.4	%	42.0-75.0
Lymphocytes	0.9	%	20.0-40.0
Monocytes	9.2	%	2.0-7.0
Neutrophils	13.1	X10^3	1.5-7.1
Lymphocytes	0.1	X10^3	0.7-4.3
Monocytes	1.3	X10^3	0.2-1.2

**Table 2:** Coagulation results

Collected	Result	Units	Reference Range
Prothrombin time (PT)	14.7	Seconds	9.3-11.9
International normalized ratio (INR)	1.42		0.87-1.13
Partial thromboplastin time (PTT)	38.6	Seconds	23.8-33.3

**Table 3:** Metabolic panel

Collected	Result	Units	Reference Range
Sodium	145	mEq/dl	136-145
Potassium	3.4	mEq/dl	3.5-5.1
Chloride	113	mEq/dl	98-107
Calcium	8.0	mg/dl	8.5-10.1
Albumin	2.7	g/dl	3.4-5.0
Lactate	1.6	mmol/l	0.4-2.0
Glucose	181	mg/dl	70-99
Blood urea nitrogen	23	mg/dl	7-18
Creatinine	1.37	mg/dl	0.55-1.02
Albumin	3.3	g/dl	3.4-5.0
Aspartate aminotransferase	11	U/L	15-37
Alanine aminotransferase	14	U/L	14-59
Sedimentation rate	30	mm/hour	0-20
Procalcitonin	6.96	ng/ml	0-1.90
C-reactive protein	2.1	mg/dl	0.0-0.2

**Table 4:** Urinalysis result

Collected	Result	Units	Reference Range
Appearance	Cloudy		clear
Color	Amber		Yellow, straw, colorless, pale yellow
Specific gravity	1.023		1.016-1.022
PH	5		5.0-7.0
Protein	+1		Negative
Bilirubin	+1		Negative
Blood	+1		Negative
Leukocytes	+1		Negative
Urine blood glucose	2.0	mg/dl	Normal, 1
White blood cells	21-50		0-2, none seen
Red blood cells	11-20		0-2, none seen
Bacteria	1+		None seen
Mucus	3+		None seen

## Discussion

The clinical manifestations of IE can range from acute and rapidly progressing to chronic with nonspecific symptoms. Fever is present in up to 90% of patients and is the most common symptom. Patients may also experience weight loss and chills [3]. According to an article by Habib et al., physicians should suspect IE in patients with a new regurgitant murmur or embolic events of an unknown origin or if there is sepsis of any unknown origin, especially if the organism is known to be associated with IE. IE should be suspected if fever is associated with the following: patients with cardiac prosthetic devices, such as implantable defibrillators or prosthetic valves, previous history of IE, previous valvular disease or congestive heart failure, immunosuppression, intravenous drug abuse, recent treatment for associated bacteremia, and blood cultures positive for IE-associated organisms or for chronic Q fever (Coxiella burnetii). Other signs of IE associated with fever are vascular or immunologic phenomena, such as emboli, Roth spots, Osler’s nodes, splinter hemorrhages, or Janeway lesions. Peripheral abscesses, such as renal, splenic, cerebral, or vertebral lesions, or a history of a pulmonary embolism should also raise suspicion of IE. Physicians should keep in mind that fever might not be present in patients that are elderly, immuno-compromised, or pre-treated with antibiotics [3].

About 20% of patients with IE also have cardiac devices or prosthetic valves. The use of the modified Duke criteria is not as sensitive in this population group. The use of an echocardiogram is not as accurate in these patients because the findings can be interpreted as complex and inconclusive. Positron-emission computed tomography (PET/CT) can be a useful addition for detecting IE in these patients [4]. Pizzi et al. performed a cohort study on the effectiveness of using PET/CT in patients who were diagnosed with suspected IE, specifically in patients with cardiac devices or prosthetic valves [4]. A total of 92 patients with a suspected prosthetic valve or cardiac-device-related IE from November 2012 to November 2014 were included. All were given an echocardiogram and a PET/CT, and their findings were compared. They found that adding PET/CT to the Duke criteria upon admission increased the sensitivity of IE. The diagnosis of possible IE cases upon admission was significantly reduced. Diagnosing patients with possible IE is a major issue in clinical practice because it can delay the management process and cause complications. By reclassifying possible IE patients into either definite or rejected IE, PET/CT could play a major role in the management and prognosis of this patient population [4].

Prompt diagnosis and treatment of invasive Candida infection are essential for an improved prognosis. However, early diagnosis is challenging. There is a Candida scoring system, which could help physicians identify patients that could benefit from early antifungal treatment. The bedside Candida scoring system equation is calculated as follows: (0.908 x total parenteral nutrition) + (0.007 x surgery) + (1.112 x multifocal Candida species colonization) + (2.038 x severe sepsis). The variables are given the value one or zero depending on whether they are present or not, respectively [5]. A study by Leroy et al. evaluated the effectiveness of this score in a cohort study of critically ill patients with Candida infection. The cohort was performed from January 2010 to March 2011 in five intensive care units (ICUs) in France. They included 94 patients who developed hospital-acquired sepsis on ICU admission or during their stay in the ICU. The incidence of invasive candidiasis was collected and the relationship with the Candida score was evaluated. They found invasive candidiasis in five of the patients. They determined that the rate of invasive candidiasis was 0% in patients with a score of two to three, 17.6% in patients with a score of four, and 50% in patients with a score of 5 (p<0.0001) [5]. Even though this study was taken in the context of ICU patients with hospital-acquired sepsis, it could still be used by physicians to screen for the Candida infection before waiting for blood culture results. 

The treatment for cases of infective endocarditis from Candida usually involves surgical removal of the infected valve, followed by an antifungal agent [6]. The antifungals used are amphotericin b or its liposomal form, with or without adding flucytosine. Amphotericin b therapy is an effective nonsurgical approach for patients with Candida infective endocarditis [7]. Monotherapy with echinocandins (such as caspofungin and micafungin) can also be used as an initial treatment, especially for patients who are at increased risk of side effects from amphotericin b [8]. Surgery is recommended in patients with prosthetic-related infective endocarditis [9]. This patient’s lesion was located on the interatrial septum and she was elderly with multiple comorbidities; which made surgical intervention difficult. Physicians should always take into consideration factors such as age and past medical history before considering surgical intervention for infective endocarditis. Patients who cannot undergo surgical procedures are recommended to receive lifelong suppression with oral fluconazole, after the initial therapy with amphotericin b or caspofungin.

Literature review

Our review of the literature revealed one similar case reported in Brazil by De Araújo et al. [article in Portuguese] [2]. Their patient was a 49-year-old male on chronic dialysis with a tunneled catheter who presented with fever and chills. He also complained of dyspnea, dry cough, and nausea. The patient had a venous catheter in the right subclavian vein. A cardiovascular examination showed no abnormalities. No other relevant information was found on physical examination. The blood cultures were positive for Candida parapsilosis. A transesophageal echocardiogram showed a mobile mass adhered to the inter-atrial septum close to the tip of the catheter. Their cardiology team decided not to perform surgery and initiated antibiotic therapy with amphotericin b instead. They then switched the patient to fluconazole. After three months of therapy, the patient was asymptomatic and showed no vegetation on transesophageal echocardiogram. Subsequently, antifungal treatment was suspended [2]. This case showed similarities in terms of diagnosis and management, except we did not use amphotericin b given our patient’s circumstances.

## Conclusions

To our knowledge, this is the first reported case of prosthetic-related endocarditis from Candida albicans on the right atrial septal wall in the United States. This patient did not present with the typical features of IE. Since she was using a subcutaneous port device, it led to the suspicion of a possible IE. This demonstrates the significance of taking a detailed patient history before ordering tests or forming a differential diagnosis. It is important for physicians to suspect IE in febrile patients who use subcutaneous port devices and promptly order a blood culture. If blood cultures are positive, removing the port device is highly recommended. Obtaining a transesophageal echocardiogram is also recommended. For patients requiring subcutaneous port devices, it is important that caregivers place the catheter tip correctly in the superior vena and confirm its location with imaging to avoid these complications. The transesophageal echocardiogram findings were critical in locating the vegetation in this patient. Physicians should avoid surgery in elderly patients with multiple comorbid conditions because it could lead to serious complications. Nonsurgical interventions with antifungal treatment have been proven effective in patients with Candida-associated infective endocarditis. The goal of presenting this case was to show that IE could occur in patients without valvular involvement. Hopefully, there could be specific recommendations in the future on how to manage IE in patients without valvular involvement.
